# Spontaneous and inducible apoptosis in oesophageal adenocarcinoma

**DOI:** 10.1054/bjoc.2001.2084

**Published:** 2001-11

**Authors:** A Raouf, D Evoy, E Carton, E Mulligan, M Griffin, E Sweeney, J V Reynolds

**Affiliations:** The Department of Clinical Surgery, Department of Histopathology, Trinity Centre for Health Sciences, Trinity College Dublin, St. James's Hospital, Dublin 8, Ireland

**Keywords:** oesophagus, adenocarcinoma, apoptosis, Ki-67, p53, bcl-2

## Abstract

The use of neoadjuvant chemoradiotherapy prior to surgery in the treatment of oesophageal adenocarcinoma has increased in recent years, and up to 25% of patients will have a complete pathological response to the neoadjuvant therapy. Many patients will not respond, however, and the knowledge of molecular factors predicting response or resistance to chemoradiotherapy is required to enhance treatment results. An understanding of apoptosis and cell proliferation may be relevant and this study focused on apoptotic indices and cell-cycle related (Ki-67, p53 and bcl-2) protein expression in a cohort of 42 patients with primary oesophageal adenocarcinoma. We documented that apoptosis occurs among viable (proliferating) tumour cells in all adenocarcinoma cases examined in this study. Pre-operative chemoradiotherapy significantly increased apoptosis and significantly decreased cell proliferation (estimated by Ki-67 expression). Immunohistochemically detected *p53* and *bcl-2* gene products had no regulatory role in the apoptotic process. The cumulative expression of p53 protein is significantly associated with increasing proliferation activity. Evaluation of apoptosis in pre-treatment specimens may have potential utility in predicting the efficacy of treatment. Assessment of the tumours proliferation activity by Ki-67 expression might identify patients who are at risk of developing metastastic disease. © 2001 Cancer Research Campaign http://www.bjcancer.com


*British Journal of Cancer* (2001) **85**(11), 1781–1786
© 2001 Cancer Research Campaign
doi: 10.1054/ bjoc.2001.2084, available online at http://www.idealibrary.com on IDEAL

### Spontaneous and inducible apoptosis in oesophageal adenocarcinoma

**A Raouf[1], D Evoy[1], E Carton[1], E Mulligan[1], M Griffin[2], E Sweeney[2] and JV Reynolds[1]**

The Department of Clinical Surgery[1] and the Department of Histopathology[2], Trinity Centre for Health Sciences, Trinity College Dublin, St. James's Hospital, Dublin 8, Ireland

**Summary** The use of neoadjuvant chemoradiotherapy prior to surgery in the treatment of oesophageal adenocarcinoma has increased in recent years, and up to 25% of patients will have a complete pathological response to the neoadjuvant therapy. Many patients will not respond, however, and the knowledge of molecular factors predicting response or resistance to chemoradiotherapy is required to enhance treatment results. An understanding of apoptosis and cell proliferation may be relevant and this study focused on apoptotic indices and cell-cycle related (Ki-67, p53 and bcl-2) protein expression in a cohort of 42 patients with primary oesophageal adenocarcinoma. We documented that apoptosis occurs among viable (proliferating) tumour cells in all adenocarcinoma cases examined in this study. Pre-operative chemoradiotherapy significantly increased apoptosis and significantly decreased cell proliferation (estimated by Ki-67 expression). Immunohistochemically detected *p53* and *bcl-2* gene products had no regulatory role in the apoptotic process. The cumulative expression of p53 protein is significantly associated with increasing proliferation activity. Evaluation of apoptosis in pre-treatment specimens may have potential utility in predicting the efficacy of treatment. Assessment of the tumours proliferation activity by Ki-67 expression might identify patients who are at risk of developing metastastic disease. © 2001 Cancer Research Campaign http://www.bjcancer.com

**Keywords**: oesophagus; adenocarcinoma; apoptosis; Ki-67; p53; bcl-2

Under normal physiological conditions and in response to chemotherapeutic drugs or irradiation, cells die by a morphologically distinct event known as apoptosis (Kerr et al, 1972; Koshiji et al, 1997). Apoptosis is a genetically regulated process that in balance with cell proliferation is required by normal tissues for remodelling, proper development and function (Kerr et al, 1972). An alteration in the activity of genes controlling the cell cycle or apoptosis can lead to pathological changes that may result in degenerative disease or cancer (Vaux et al, 1988; Lane, 1992; Katada et al, 1997).

Both *p53* and *bcl-2* gene products are involved in the regulation of cell cycle and apoptosis. The *p53* tumour suppressor gene encodes a 53 kDa nuclear phosphoprotein. This protein functions as a nuclear transcription factor and activates other genes involved in growth arrest and apoptosis in response to DNA damage and other cellular insults (Lane, 1992; Leland and Kastan, 1994; Kagawa et al, 1997; Levin, 1997). The loss of functional wild-type p53 protein due to mutation or allelic deletion of the gene significantly alters suppressor activity and increases genomic instability (Lane, 1992; Leland and Kastan, 1994; Paules et al, 1995). The bcl-2 oncogene encodes a 25 kDa integral membrane protein that is involved in the regulation of programmed cell death by inhibiting apoptosis (Hockenbery et al, 1990). Bcl-2 protein allows cells to accumulate by prolonging their life span (Hockenbery et al, 1990; Liu et al, 1991) and this effect may contribute to neoplastic development (Vaux et al, 1988). Studies have demonstrated that high levels of bcl-2 protect cells from apoptosis induced by a variety of chemotherapeutic agents (Inada et al, 1997; Miyake et al, 1998; Zhang et al, 1999) and irradiation (Rupnow et al, 1998). The precise biochemical function of bcl-2 is still under investigation and it is likely that the inter-relationship between bcl-2 family proteins and their relative ratios will eventually determine cell susceptibility to undergo apoptosis or survive after an apoptotic signal (Oltvai et al, 1993; Reed, 1994; Sato et al, 1994).

The Oesophageal Unit in St James's Hospital and Trinity College Dublin has reported in a randomised trial that neaodjuvant chemoradiotherapy prior to resectional surgery results in a pathological complete response rate of 25% and improved survival compared to surgery alone (Walsh et al, 1996). Approximately 60% of patients have no response to neoadjuvant therapy. There is a need to identify molecular factors predicting response or resistance to this treatment. The purpose of this study was 2-fold: first, to investigate the frequency of apoptosis and cell proliferation in oesophageal adenocarcinoma and the influence of p53 and bcl-2, and secondly, to determine the influence of neoadjuvent chemoradiotherapy on these parameters.

*Received 19 October 2000*
*Revised 17 July 2001*
*Accepted 19 July 2001*

*Correspondence to:* JV Reynolds

### MATERIALS AND METHODS

### Patients

Archival formalin-fixed paraffin-embedded tissue samples from 42 patients (32 male, 10 female) with primary oesophageal adenocarcinoma was included in this study. Patient age ranged from 48 to 77 years (64.3 ± 7.6 mean ± SD, 66 median). All patients had oesophagogastro-endoscopy and histological confirmation of diagnosis prior to treatment with chemotherapy (cisplatin and 5-fluorouracil) and radiotherapy (CRX) followed by oesophagectomy. Surgery was undertaken at a median of 4 (range 3–6) weeks following neoadjuvant therapy. All patients were treated with curative intent and patients with T4 disease or metastatic disease were excluded.

### Histopathological examination

Diagnostic biopsy and resection specimens were fixed in 10% buffered formalin, routinely processed and embedded in paraffin. Haematoxylin and eosin slides were reviewed and sequential 4 µm sections were used for immunohistochemistry. Tumours were staged according to the TNM system. Patients were defined as complete pathological response (CPR) to CRX when no microscopic tumour cells were identified at resection ($n$ = 9). Patients ($n$ = 29) were defined as non-responders (NPR) to CRX when there was no evidence of pathological response in the resection specimen e.g. the presence of lymph node metastasis, lymphovascular permeation and no down-staging (i.e. pT3) compared to pre-treatment status. A third intermediate group ($n$ = 4) was defined as having a major pathological response (MPR) to CRX. These patients were all lymph node-free and had microscopic residual tumour cells only in any part of the oesophageal wall. For the purpose of statistical analysis CPR and MPR data are combined to one group ($n$ = 13) versus NPR ($n$ = 29).

### Assessment of apoptosis

In situ terminal deoxynucleotide transferase (TdT) method was used for identification of apoptotic cells and bodies in paraffin tissue sections using Apoptag® peroxidase in situ apoptosis detection kit (S7100) (Intergen Company). Following the manufacturer's instructions 4 µm sections were deparaffinised, rehydrated and placed in 10 mM citrate buffer pH at 6.0 and gently boiled for 10 min in a microwave oven. Sections were incubated with 20 µg ml$^{-1}$ proteinase K at room temperature for 10 min followed by exposure to 3% hydrogen peroxide in PBS to quench endogenous peroxidase activity. The sections were then treated with terminal transferase enzyme and digoxigenin-labelled nucleotides. Apoptotically fragmented DNA that have been labelled with the digoxigenin nucleotide was then allowed to bind an anti-digoxigen antibody that is conjugated to peroxidase molecules. The reaction products of peroxidase were visualised with 3′-3′ diaminobenzidine (DAB). After the color reaction, sections were counterstained with methyl green. Unstained rat mammary glands (Apoptag® positive control slides (S7115), Intergen Company) were used as positive controls. Omission of the TdT during the staining procedure provided negative controls. The number of apoptotic cells and bodies in a total of 2000 tumour cells were counted and the percentage positivity recorded as the apoptotic index (AI).

### Immunohistochemistry

Sections of the same part of the tumour used for detection of apoptotic cells were used to assess Ki-67, p53 and bcl-2 immunoreactivity by standard avidin–biotin complex method. Sections were incubated with polyclonal anti-Ki-67, monoclonal mouse anti-p53-DO7 and anti-bcl-2 clone 124, (Dako A/S, Denmark) dilution 1:50–overnight at 4˚C. Biotinylated swine anti-rabbit and rabbit anti-mouse (1:300 dilution–30 min) were used as a secondary antibody. The colour reaction product was obtained with DAB and a haematoxylin counterstained. Sections of human tonsil, colon adenocarcinoma and follicular lymphoma known to be positive for ki-67, p53 and bcl-2 were used as positive controls. Omission of the primary antibody provided negative controls. Out of 2000 tumour cells the number showing moderate to intense immunostaining was counted and the percentage positivity recorded as the labelling index (LI). Tumours demonstrating weak immunopositivity with LI < 5% were considered to be negative for p53 or bcl-2.

### Statistical analysis

All statistical calculations were carried out with the StatView software package (Abacus Concepts, Berkeley, CA). The significance of association among variables (AI, Ki-67 LI, p53 and bcl-2 expression) and the clinicopathological factors including tumour differentiation, depth of invasion and lymph node status was determined by Mann–Whitney or Kruskal–Wallis tests. The association between p53, bcl-2 expression and type of response to CRX was determined by Fisher exact test. For paired comparison of AI and Ki-67 LI pre- to post-CRX values Wilcoxon test was used. Statistical significance was defined as $P < 0.05$.

### RESULTS

### Apoptotic index, Ki-67 labelling index, p53 and bcl-2 expression in oesophageal adenocarcinomas prior to chemoradiotherapy (Table 1)

Apoptotic cells and bodies were detected among viable tumour cells in all adenocarcinoma cases examined in this study. The

**Table 1** Apoptotic index, Ki-67 labelling index, p53 and bcl-2 expression in oesophageal adenocarcinomas prior to chemoradiotherapy

| Parameters | No. of cases | Apoptotic index Mean ± SD/Median | *P* value | Ki-67 labelling index Mean ± SD/Median | *P* value |
|---|---|---|---|---|---|
| Response to CRX | | | | | |
| CPR and MPR | 13 | 0.83 ± 0.31/0.75 | | 35.1±15.7/33.7 | |
| NPR | 29 | 0.60 ± 0.29/0.55 | 0.01 | 38.8±12.0/37.1 | 0.35 |
| p53 expression | | | | | |
| negative tumours | 15 | 0.57 ± 0.34/0.52 | | 31.9±7.8/35.2 | |
| positive tumours | 27 | 0.73 ± 0.29/0.65 | 0.07 | 40.9±14.5/41.9 | 0.01 |
| Bcl-2 expression | | | | | |
| negative tumours | 33 | 0.67 ± 0.32/0.64 | | 37.5±13.8/37.2 | |
| positive tumours | 9 | 0.68 ± 0.32/0.56 | 0.79 | 38.3±10.7/36.0 | 0.80 |
| Tumour differentiation | | | | | |
| well/moderate | 30 | 0.64 ± 0.29/0.57 | | 38.3±12.6/36.7 | |
| poor | 12 | 0.74 ± 0.36/0.71 | 0.26 | 36.0±15.4/34.6 | 0.48 |

*© 2001 Cancer Research Campaign*

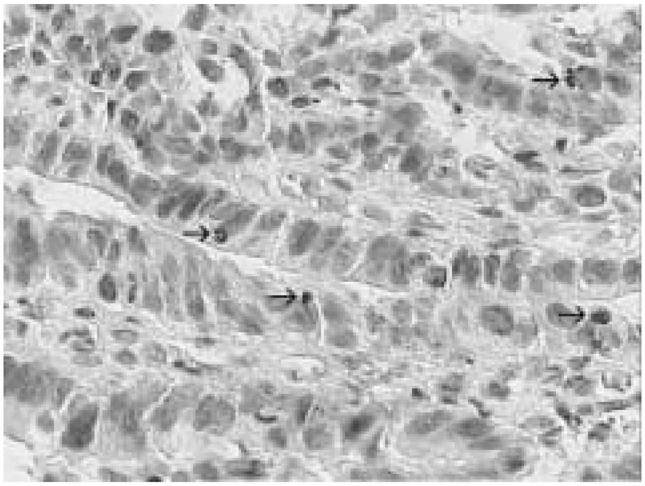

**Figure 1** In situ labelling of apoptotic tumour cells; oesophageal adenocarcinoma pre-treatment. This patient had a complete pathological response to chemoradiotherapy. Apoptotic tumour cells are characterised by condensed chromatin and nuclear fragmentation represented by dark black staining (arrows, magnification ×400). The apoptotic index is significantly higher in chemoradio-sensitive compare to -resistant tumours

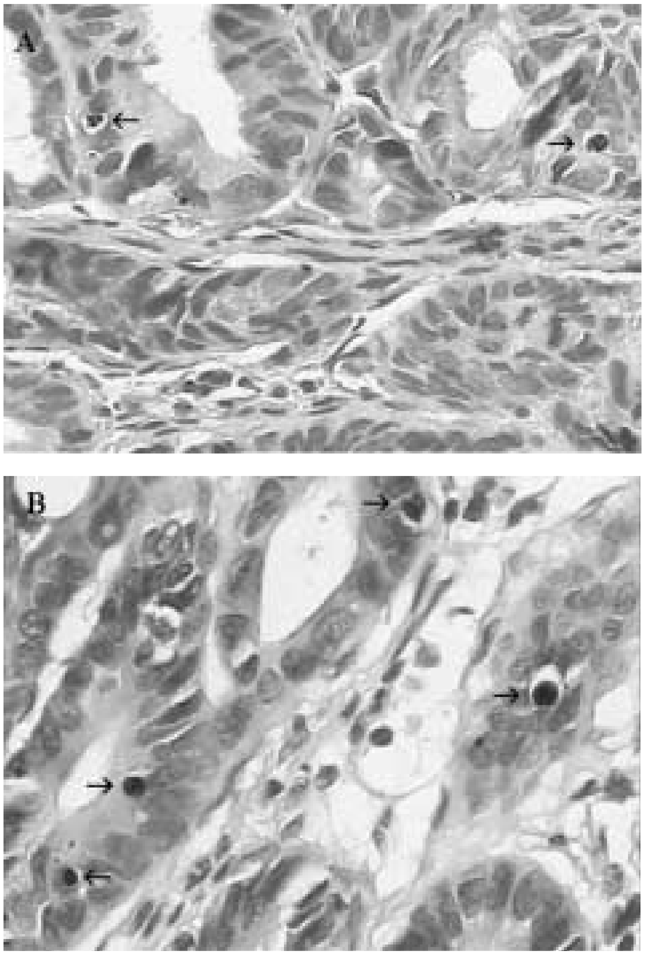

**Figure 2** In situ labelling of apoptotic tumour cells of a patient with oesophageal adenocarcioma: (**A**) prior to; (**B**) after chemoradiotherapy. This patient showed no pathological response to neoadjuvant therapy. Apoptotic tumour cells are characterised by condensed chromatin and nuclear fragmentation represented by dark black staining (arrows, magnification (**A**) ×250 and (**B**) ×400). Apoptotic index is significantly higher in resected tumour following chemoradiotherapy occurrence of spontaneous apoptosis in pre-CRX tumours ($n$ = 42) ranged from 0.16 to 1.60% (0.68 ± 0.32 mean ± SD; 0.63 median). The proliferation index, assessed by Ki-67 LI, ranged from 2 to 64.8% (38.1 ± 13.2 mean ± SD; 37.1 median). p53 and bcl-2 protein expression was detected in 64.2% (27/42) and 21.4% (9/42) of tumours, respectively.

A significant ($P$ = 0.01) association was observed between the apoptotic index and the type of response to CRX. Tumours that responded completely to preoperative CRX and those that showing a major pathological response had a higher apoptotic index in pre-treatment tumour samples compared to tumours with no evidence of pathological response (Figures 1 and 2A). There was no association between the tumour response or resistance to CRX and Ki-67 expression. There was no significant association between the apoptotic index and p53 or bcl-2 expression. Accumulation of p53 protein in tumour samples was significantly associated with high Ki-67 expression ($P$ = 0.01).

### Expression of p53 and bcl-2 and type of response to chemoradiotherapy (Table 2)

Pre-treatment tumour samples ($n$ = 9) of the subsequent complete pathological responders to CRX demonstrated 77.7% (7/9) and 11.1% (1/9) p53 and bcl-2 immunopositivity, respectively. Pre-treatment tumour samples ($n$ = 4) of the subsequent major pathological responders were 50% (2/4) p53 and 50% (2/4) p53 and 50% (2/4) bcl-2 immunopositive, respectively. Tumours ($n$ = 29) that did not respond to CRX expressed p53 in 62% (18/29) of cases and bcl-2 in 20.6% (6/29). Expression of p53 or bcl-2 did not statistically associate with tumour response or resistance to CRX.

### Apoptotic index, Ki-67 labelling index, p53 and bcl-2 expression in oesophageal adenocarcinoma after chemoradiotherapy (Table 3)

It was not possible to determine the apoptotic or proliferation indices in residual carcinomas of patients having a major pathological response to CRX ($n$ = 4) due to an insufficient number of residual tumour cells. Expression of p53 and bcl-2 was detected in 25% (1/4) of residual tumours. In residual carcinomas of patients having no pathological response to CRX ($n$ = 29) the apoptotic index was significantly increased by CRX compared to its pre-treatment value ($P$ < 0.0001) (Figure 2B), and the proliferation index (Ki-67 LI) was significantly decreased ($P$ = 0.0003). The expression of p53 and bcl-2 was detected in 79.3% (23/29) and 24.1% (7/29) of tumours, respectively. Neither p53 nor bcl-2 expression was statistically associated with the apoptotic or proliferation index.

**Table 2** Expression of p53 and bcl-2 and type of response to chemoradiotherapy

| Parameters | CPR and MPR | NPR | *P* value |
|---|---|---|---|
| P53 expression | | | |
| negative tumours | 4 | 11 | |
| positive tumours | 9 | 18 | 0.73 |
| Bcl-2 expression | | | |
| negative tumours | 10 | 23 | |
| positive tumours | 3 | 6 | 0.9 |

© 2001 Cancer Research Campaign

**Table 3** Apoptotic index, Ki-67 labelling index, p53 and bcl-2 expression in oesophageal adenocarcinoma resection specimens in patients who did not respond to chemoradiotherapy

| Parameters | No. of cases | Apoptotic index Mean ± SD/Median | *P* value | Ki-67 labelling index Mean ± SD/Median | *P* value |
|---|---|---|---|---|---|
| Labelling index | | | | | |
| pre-CRX | 29 | 0.60 ± 0.29/0.55 | | 38.8 ± 12.0/37.1 | |
| post-CRX | 29 | 1.36 ± 0.47/1.40 | < 0.0001 | 24.7 ± 14.1/26.5 | 0.0003 |
| p53 expression | | | | | |
| negative tumours | 6 | 1.33 ± 0.50/1.20 | | 19.0 ± 14.3/18.7 | |
| positive tumours | 23 | 1.37 ± 0.47/1.48 | 0.78 | 26.2 ± 14.0/27.1 | 0.45 |
| Bcl-2 expression | | | | | |
| negative tumours | 22 | 1.40 ± 0.48/1.44 | | 25.6 ± 14.8/26.4 | |
| positive tumours | 7 | 1.20 ± 0.44/1.31 | 0.62 | 22.1 ± 12.7/27.3 | 0.87 |
| Tumours differentiation | | | | | |
| well/moderate | 25 | 1.35 ± 0.50/1.31 | | 23.8 ± 15.0/26.3 | |
| poor | 4 | 1.40 ± 0.18/1.40 | 0.75 | 30.4 ± 4.5/31.0 | 0.18 |
| Tumours extension (pT) | | | | | |
| T1 | 5 | 0.90 ± 0.24/0.91 | | 27.2 ± 7.1/27.1 | |
| T2 | 3 | 1.29 ± 0.19/1.30 | | 41.8 ± 15.4/36.8 | |
| T3 | 21 | 1.48 ± 0.47/1.50 | 0.02 | 21.7 ± 13.9/24.3 | 0.06 |
| Lymph node metastasis (pN) | | | | | |
| negative | 18 | 1.39 ± 0.51/1.40 | | 20.5 ± 13.8/22.5 | |
| positive | 11 | 1.31 ± 0.40/1.20 | 0.70 | 31.7 ± 12.2/31.6 | 0.008 |

CRX = pre-operative chemoradiotherapy; CPR = complete pathological response to CRX; MPR = major pathological response to CRX; NPR = no pathological response to CRX; *P* value < 0.05 considered to be significant.

### Apoptotic index, Ki-67 labelling index, p53 and bcl-2 expression and clinicopathological parameters

Both before and after CRX, no significant association was observed between the apoptotic or proliferation index and tumour differentiation. Post-CRX, increasing depth of invasion (pT) of residual tumours was associated with an increasing apoptotic index ($P = 0.02$) and a high Ki-67 expression was significantly associated with the presence of lymph node metastasis ($P = 0.008$).

### DISCUSSION

Neoadjuvant chemotherapy with and without radiotherapy can result in complete or major pathological responses and improve local control and overall survival of patients with cancer (Walsh et al, 1996; Giatromanolaki et al, 1999; Kollmannsberger et al, 2000). The process of programmed cell death is considered a mechanism to counter proliferation activity (Katada et al, 1997) and is a potential predictor of treatment efficacy (Scott et al, 1998; Logsdon et al, 1999; Cameron et al, 2000; Rodel et al, 2000). In the present study the degree of apoptosis was analysed in relation to clinicopathological parameters, cell proliferation activity, and the expression of apoptotic/cell-cycle-related proteins.

Apoptotic tumour cells were visualised by enzymatic labelling of fragmented DNA with a terminal transferase reaction. The methodology facilitates detection of early and late stages of the apoptotic cells in tissue sections. The results from this study indicate that an increased rate of spontaneous apoptosis in oesophageal adenocarcinomas may be a prognostic biomarker for chemoradiotherapy in patients with oesophageal adenocarcinoma. Tumours with a high apoptotic index were more sensitive to chemotherapy and radiation therapy than tumours with a low apoptotic rate, with more complete and major responders to the regimen in the high apoptosis group. The combination of chemotherapy and radiation therapy induces apoptosis. This was evident from analysis of resection specimens in patients who failed to respond to neoadjuvant therapy. In these patients, the apoptotic index in 23 of 29 tumours was more than twice its pre-treatment value. The remaining tumours showed less of an increase in the apoptotic index (4 cases) or slight reduction in the percentage of apoptotic cells (2 cases). The pattern of change in apoptosis was unavailable from patients who had a complete or major response to therapy, and this is the subject of the current study.

The biological and molecular response of tumours to neoadjuvant therapy is not well elucidated. Pre-operative chemoradiotherapy may modulate tumour growth via induction of apoptosis and overcome the adverse influence of the biological factors or genes that usually down-regulate apoptosis. Recent studies indicate that different types of tumour cells undergo programmed cell death via different pathways. Both p53 (Kagawa et al, 1997) and bcl-2 (Inada et al, 1997; Miyake et al, 1998; Luo et al, 1999) have been shown to be key regulators of this process. However, occurrence of apoptosis and its modulation via p53 or bcl-2-independent pathways has also been reported (Xie et al, 1999; Kupryjanczk et al, 2000). In oesophageal adenocarcinoma the latter scenario is most likely. We found, for instance, that tumours over-expressing p53 (before and after chemoradiotherapy) had similar apoptotic indices to p53-negative tumours. It is possible that immunohistochemically detected p53 might be associated with an accumulation of functionless protein due to *p53* gene mutation. As one of the normal functions of p53 is to control the cell cycle and apoptosis in response to DNA damage such as that induced by chemotherapeutic agents or irradiation, tumour cells with defective p53 might be less able to repair DNA damage or to undergo apoptosis. The

 *© 2001 Cancer Research Campaign*

heterogeneity of bcl-2 expression in tumour cells was noted in contrast to p53 which conforms to a homogenous pattern. An absence of a direct correlation between p53 and bcl-2 expression was also observed. Moreover, the histological differentiation or stage of the tumour was not a major determinant of apoptosis, a feature in contrast to recent reports of ovarian, tongue and colorectal carcinomas (Sugamura et al, 1998; Xie et al, 1999; Kupryjanczk et al, 2000).

Many studies have investigated the link between p53 overexpression and tumour growth. Some indicated a positive relationship (Augustin et al, 1997; Ikeguchi et al, 1997; Ozer et al, 1998) and other have failed to establish such an association (Slootweg et al, 1994; Suto et al, 1998). Ki-67 is a nuclear protein expressed throughout the cell cycle except in the resting phase ($G_0$) (Gerdes et al, 1984), and therefore is widely used as a growth marker to evaluate the proliferation activity of the normal or neoplastic cell population. In addition, the level of expression of p53 and Ki-67 together Augustin et al, 1997 or independently (Suto et al, 1998) has been found to correlate well with many clinical and pathological variables. The data presented here indicate the presence of a significant association between p53 and Ki-67 expression. The cumulative expression of p53 in tumour biopsy samples was associated with an increasing proliferative index as measured by Ki-67 labelling index. The observed correlation between p53 and Ki-67 no longer exists in the resected tumours due to significant decrease in proliferative index following chemoradiotherapy, while, the expression level of p53 is largely unchanged. The expression of p53 and Ki-67 did not impact as predictors of response or resistance to neoadjuvant therapy. Ki-67 expression may, however, relate to metastatic potential as it correlated with nodal status in resected specimens, an observation also reported for other tumours (Ikeguchi et al, 1997; Suto et al, 1998).

In conclusion, this study demonstrates that apoptosis occurs among viable tumour cells in all adenocarcinoma cases examined. Pre-operative chemoradiotherapy significantly increased apoptotic cell death and significantly decreased cell proliferation rate. Immunohistochemically detected *p53* and *bcl-2* gene products have no regulatory role in the apoptotic process in oesophageal adenocarcinoma, nor did they predict response or resistance to the neoadjuvant regimen. In contrast, a high pre-treatment apoptotic index correlates with response to neoadjuvant therapy. The evaluation of apoptosis in pre-treatment specimens may have potential application in predicting the efficacy of neadjuvant approaches for oesophageal adenocarcinoma.

### REFERENCES

Cameron DA, Keen JC, Dixon JM, Bellamy C, Hanby A, Anderson TJ and Miller WR (2000) Effective tamoxifen therapy of breast cancer involves both antiproliferative and pro-apoptotic changes. *Eur J Cancer* **36**: 845–851

Gerdes J, Lemke H, Baisch H, Wacker HH, Schwab U and Stein H (1984) Cell cycle analysis of a cell proliferation-associated human nuclear antigen defined by the monoclonal antibody Ki-67. *J Immunology* **133**: 1710–1715

Giatromanolaki A, Koukourakis MI, Georgoulias V, Gatter KC, Harris AL and Fountzilas G (1999) Angiogenesis vs. response after combined chemoradiotherapy of squamous cell head and neck cancer. *Int J Cancer* **80**: 810–817

Hockenbery D, Nunez G, Milliman C, Schreiber RD and Korsmeyer SJ (1990) Bcl-2 is an inner mitochondrial membrane protein that blocks programmed cell death. *Nature* **348**: 334–336

Ikeguchi M, Saito H, Katano K, Tsujitani S, Maeta M and Kaibara N (1997) Clinicopathologic Significance of the Expression of Mutated p53 Protein and the proliferative activity of cancer Cells in Patients with Esophageal Squamous Cell Carcinoma. *J Am Coll Surg* **185**: 398–403

Inada T, Ichikawa A, Igarashi S, Kubota T and Ogata Y (1997) Effect of preoperative 5-fluorouracil on apoptosis of advanced gastric cancer. *J Surg Oncol* **65**: 106–110

Kagawa S, Fujiwara T, Hizuta A, Yasuda T, Zhang WW, Roth JA and Tanaka N (1997) p53 expression overcomes p21WAF1/CIP1-mediated G1 arrest and induced apoptosis in human cancer cells. *Oncogene* **15**: 1903–1909

Katada N, Hinder RA, Smyrk TC, Hirabayashi N, Perdikis G, Lund RJ, Woodward T and Klingler PJ (1997) Apoptosis is inhibited early in the dysplasia-carcinoma sequence of Barrett esophagus. *Arch Surg* **132**: 728–733

Kerr JFR, Wyllie AH and Currie AR (1972) Apoptosis: a basic biological phenomenon with wide-ranging implication in tissue kinetics. *Br J Cancer* **26**: 239–257

Kollmannsberger C, Quietzch D, Haag C, Lingenfelser T, Schroeder M, Hartmann JT, Baronius W, Hempel V, Clemens M, Kanz L and Bokemeyer C (2000) A phase II study of paclitaxel, weekly, 24-hour continous infusion 5-fluorouracil, folinic acid and cisplatin in patients with advanced gastric cancer. *Br J Cancer* **83**: 458–462

Koshiji M, Adachi Y, Taketani S, Takeuchi K, Hioki K and Ikehara S (1997) Mechanism underlying apoptosis induced by combination of 5-fluorouracil and interferon-gamma. *Biochem Biophys Res Commun* **52**: 376–381

Kupryjanczky J, Dansonka MA, Szymanska T, Karpinska G, Rembiszewska A, Rusin M, Konopinski R, Kraszewska E, Timorek A, Yandell DW and Stelmachow J (2000) Spontaneous apoptosis in ovarian carcinomas: a positive association with p53 gene mutation is dependent on growth fraction. *Br J Cancer* **82**: 579–583

Lane DP (1992) p53, guardian of the genome. *Nature* **358**: 15–16

Leland HH and Kastan MB (1994) Cell cycle control and cancer. *Science* **266**: 1821–1828

Levin AJ (1997) p53, the cellular gatekeeper for growth and division. *Cell* **88**: 323–331

Liu YJ, Mason DY, Johnson GD, Abbot S, Gregory CD, Hardie DL, Gordon J and MacLennan IC (1991) Germinal center cells express bcl-2 protein after activation by signals which prevent their entry into apoptosis. *Eur J Immunol* **21**: 1905–1910

Longsdon MD, Meyn RE Jr., Besa PC, Pugh WC, Stephens LC, Peter LJ, Milas L, Cox JD, Cabanillas F, Brisbay S, Andersen M and McDonnell TJ (1999) Apoptosis and the bcl-2 gene family – patterns of expression and prognostic value in stage I and II follicular center lymphoma. *Int J Radiat Oncol Biol Phys* **44**: 19–29

Luo D, Cheng CS and Xie Y (1999) Expression of bcl-2 family proteins during chemotherapeutic agents-induced apoptosis in the hepatoblastoma HepG2 cell line. *Br J Bio Science* **56**: 114–122

Miyake H, Hanada N, Nakamura H, Kagawa S, Fujiwara T, Hara I, Eto H, Gohji K, Arakawa S, Kamidono S and Saya H (1998) Overexpression of bcl-2 in bladder cancer cells inhibits apoptosis induced by cisplatin and adenoviral-medioated p53 gene transfer. *Oncogene* **16**: 933–943

Oltvai ZN, Milliman CL and Korsmeyer SJ (1993) Bcl-2 heterodimerizes in vivo with a conserved homology, Bax, that accelerates programmed cell death. *Cell* **74**: 609–619

Ozer E, Canda T and Kuyucuodlu F (1998) p53 mutations in bilateral breast carcinoma. Correlation with Ki-67 expression and the mean nuclear volume. *Cancer Lett* **122**: 101–106

Paules RS, Levedakou EN, Wilson SJ, Innes CL, Rhodes N, Tlsty TD, Galloway DA, Donehower LA, Tainsky MA and Kaufmann WK (1995) Defective G2 checkpoint function in cells from individuals with familial cancer syndromes. *Cancer Res* **55**: 1763–1773

Reed JC (1994) Bcl-2 and the regulation of programmed cell death. *J Cell Biol* **124**: 1–6

Rey A, Lara PC, Redondo E, Valdés E and Apolinario R (1997) Overexpression of p53 in Transitional Cell Carcinoma of the Renal Pelvis and Ureter: relation to Tumor Proliferation and Survival. *Cancer* **79**: 2178–2185

Rodel C, Grabenbauer GG, Rodel F, Birkenhake S, Kuhn R, Martus P, Zorcher T, Fursich D, Papadopoulos T, Dunst J, Schrott KM and Sauer R (2000) Apoptosis, p53, bcl-2 and Ki-67 in invasive bladder carcinoma: possible predictors for response to radiochemotherapy and successful bladder preservation. *Int J Radiat Oncol Biol Phys* **46**: 1213–1221

Rupnow BA, Murtha AD, Alarcon RM, Giaccia AJ and Knox SJ (1998) Direct evidence that apoptosis enhances tumour responses to fractionated radiotherapy. *Cancer Res* **58**: 1779–1784

Sato T, Hanada M, Bodrug S, Irie S, Iwama N, Boise LH, Thompson CB, Golemis E, Fong L, Wang HG and Reed JC (1994) Interaction among members of the Bcl-2 protein family analyzed with a yeast two-hybrid system. *Proc Natl Acad Sci USA* **91**: 9238–9242

Scott N, Hale A, Deakin M, Hand P, Adab FA, Hall C, Williams GT and Elder JB (1998) A histopathological assessment of the response or

*© 2001 Cancer Research Campaign*

rectal adenocarcinoma to combination chemo-radiotherapy: relationship to apoptotic activity, p53 and bcl-2 expression. *Eur J Surg Oncol* **24**: 169–173

Slootweg PJ, Koole R and Hordijk GJ (1994) The presence of p53 protein in relation to Ki-67 as cellular proliferation marker in head and neck squamous cell carcinoma and adjacent dysplastic mucosa. *Eur J Cancer B Oral Oncol* **30B**: 138–141

Sugamura K, Makino M and Kaibara N (1998) Apoptosis as a prognostic factor in colorectal carcinoma. *Surg Today* **28**: 145–150

Suto T, Sugai T, Nakamura S, Funato O, Nitta H, Sasaki R, Kanno S and Saito K (1998) Assessment of the Expression of p53, MIB-1 (KI-67 Antigen), and Argyrophilic Nucleolar Organizer Regions in Carcinoma of the Extrahepatic Bile Duct. *Cancer* **82**: 86–95

Vaux DL, Cory S and Adams JM (1988) Bcl-2 gene promotes haemopoietic cell survival and cooperates with c-myc to immortalize pre-B cells. *Nature* **335**: 440–442

Walsh TN, Noonan N, Hollywood D, Kelly A, Keeling N and Hennessy TPJ (1996) A comparison of multimodal therapy and surgery for esophageal adenocarcinoma. *The New England Journal of Medicine* **335**: 462–467

Xie X, Clausen OP, De Angelis P and Boysen M (1999) The proqnostic value of spontaneous apoptosis, bax, bcl-2, and p53 in oral squamous cell carcinoma of the tongue. *Cancer* **86**: 913–920

Zhang GJ, Kimijima I, Onda M, Kanno M, Sato H, Watanabe T, Tsuchiya A, Abe R and Takenoshita S (1999) Tamoxifen-induced apoptosis in breast cancer cells relates to down-regulation of bcl-2, but not bax and bcl-X(L), without alteration of p53 protein levels. *Clin Cancer Res* **5**: 2971–2977

*© 2001 Cancer Research Campaign*
